# Revisiting a Sample of U.S. Billionaires: How Sample Selection and Timing of Maternal Condition Influence Findings on the Trivers-Willard Effect

**DOI:** 10.1371/journal.pone.0057446

**Published:** 2013-02-21

**Authors:** Sebastian Schnettler

**Affiliations:** Department of Sociology, University of Konstanz, Konstanz, Germany; CNRS, Université de Bourgogne, France

## Abstract

Based on evolutionary theory, Trivers & Willard (TW) predicted the existence of mechanisms that lead parents with high levels of resources to bias offspring sex composition to favor sons and parents with low levels of resources to favor daughters. This hypothesis has been tested in samples of wealthy individuals but with mixed results. Here, I argue that both sample selection due to a high number of missing cases and a lacking specification of the timing of wealth accumulation contribute to this equivocal pattern. This study improves on both issues: First, analyses are based on a data set of U.S. billionaires with near-complete information on the sex of offspring. Second, subgroups of billionaires are distinguished according to the timing when they acquired their wealth. Informed by recent insights on the timing of a potential TW effect in animal studies, I state two hypotheses. First, billionaires have a higher share of male offspring than the general population. Second, this effect is larger for heirs and heiresses who are wealthy at the time of conception of all of their children than for self-made billionaires who acquired their wealth during their adult lives, that is, after some or all of their children have already been conceived. Results do not support the first hypothesis for all subgroups of billionaires. But for males, results are weakly consistent with the second hypothesis: Heirs but not self-made billionaires have a higher share of male offspring than the U.S. population. Heiresses, on the other hand, have a much lower share of male offspring than the U.S. average. This hints to a possible interplay of at least two mechanisms affecting sex composition. Implications for future research that would allow disentangling the distinct mechanisms are discussed.

## Introduction

Trivers and Willard predicted an association between maternal conditions and sex ratios at birth [1]. They based this prediction on inclusive fitness theory and research on population sex ratios and proposed the following hypothesis: Parents' reproductive success should be higher if they favored sons when in good condition and daughters when in bad condition. This should hold true if (a) the condition of offspring correlates with mother's condition during pregnancy or through investment after birth and (b) if parental condition influences the reproductive success of sons more strongly than that of daughters. In species with high paternal investment (e.g., humans), the difference between the sexes in cost for reproduction and thus the expected differences in variance of reproductive success are relatively low [1], [2]. Whereas conditions (a) and (b) find support from a variety of sources even for humans in contemporary societies [3]–[7], it has been argued by evolutionary psychologists that the existence of a link between socioeconomic status and fertility is not a necessary precondition for a Trivers-Willard (TW) effect to occur in contemporary societies – if it held for long enough in our evolutionary, ancestral environment [8], [9].

In human populations, a sex ratio of about 105–106 boys for every 100 girls is considered ‘natural’ [10]. Whereas in a number of historical populations prior to the demographic transition, sex ratios differed markedly from this natural sex ratio for status-based subgroups [7], [11], [12], empirical evidence in support of the TW hypothesis for humans in contemporary developed societies remains inconclusive [13]. Results are usually based on two types of samples with distinct, but different advantages. The first group of samples covers the general population and obtains estimates that are representative of the overall population. Here, survey studies show mixed results [8], [14], [15], but they also lack statistical power to detect small expected effect sizes [16]. Large-scale population registers, on the other hand, reveal statistically significant, yet very small TW-consistent sex-ratio biases [17], [18]. The second group of studies focuses on individuals at the upper end of the wealth distribution of a society, individuals that are often not reliably represented in general surveys. Because for these samples expected effect sizes are large [19], these studies can be informative despite their low case numbers, and allow one to assess the upper limit of the TW effect. Existing empirical studies on the wealthy report mixed results, ranging from null-effects to very large TW-consistent effects [19]–[23].

The mixed empirical pattern may be the result of data limitations in previous studies. Therefore, this study improves two central issues: First, I use an improved data set with a much lower rate of missing information than previous studies and minimize sample selection bias in the estimate of the TW effect. For two reasons male offspring is more likely to appear in newspaper or Web searches than female offspring: Females more often than males change their last names upon marriage and, given a persisting ‘glass ceiling’ effect [24], women in the US are still less likely than men to reach high-status positions in the occupational hierarchy. If the percentage of missing information regarding the sex of billionaires' offspring is high, an apparent male-biased sex ratio could be an artifact. It would be a result from sample selection and not a real effect resulting from a hypothesized TW mechanism. This is a problem that has been neglected in previous studies using high-wealth samples [19], [20]. For the data analyzed in this paper, the problem of selection bias was minimized by applying a very meticulous search strategy that yielded near-complete child information for the sample of billionaires.

Following-up on recent advances in research on the TW effect among animals, the second issue to be improved is to provide a better specification of the timing of wealth accumulation relative to the timing of conception of a child. This allows me to derive important differences in the TW effect between subgroups of the wealthy. Similar to studies on human samples, studies examining the TW effect among animals often rely on correlational evidence and report mixed findings. Potential small-sample and publication biases have prompted researchers to ask whether the TW effect may, in fact, be an artifact [2], [25]–[27]. However, recent advances on the timing of a possible TW mechanism may explain at least part of the mixed empirical pattern. From a theoretical perspective, a potential TW mechanism may operate at various stages, e.g., by affecting survival or fertilization probabilities of X- and Y-chromosome-carrying spermatozoa, by differential vulnerability of male or female fetuses during pregnancy, or by means of sex-selective parental investment post-partum [10]. From an evolutionary perspective, costs associated with the respective mechanism should be minimized in order to maximize reproductive fitness. Therefore, the earlier a sex-selective mechanism gets activated, the less somatic energy has to be written off as sunk fitness costs. Consistent with this theoretical reasoning, a meta-analysis of studies examining the TW effect in mammals shows support for the TW effect when the timing of measurement of maternal condition is taken into account: ‘When body condition, weight or food are measured or manipulated around conception, 74% of studies support the TW [hypothesis], whereas measures taken during gestation (41% support) or at birth (5%) show little relationship with sex ratio’ [28]. Research on mechanisms has further drawn attention to a possible role of paternal and particularly maternal hormone concentrations close to conception [29]–[32].

In studies on the TW effect in human populations, maternal condition, operationalized as socioeconomic status [1], is often measured at one particular date either years before or after conception for all people in the sample, without systematic variation that would allow one to detect timing effects. In order to fill this gap and to analyze the role of timing, I revisit data on the richest 400 US Americans [19], amended with information on whether wealth was inherited or self-made, an information that allows one to distinguish the intergenerational timing of wealth accumulation: A much higher percentage of heirs will already (know to) be wealthy when conceiving their first and all subsequent children, whereas self-made billionaires may not have achieved high-wealth status upon conception of at least some of their children. This leads to the following two hypotheses: First, I expect sex ratios to be higher than the population average in all billionaire subgroups. Second, sex ratios should be more strongly male-biased among individuals who inherited their wealth than among self-made billionaires who achieved their wealth status during their adult lives.

## Data & Methods

The sample is based on a list of the 400 richest US Americans published by Forbes Magazine in 2009 [33]. This list includes information on estimated individual net worth of listees [34], their wealth rank among the Forbes 400, and whether net worth is considered self-made or inherited. In addition, the Forbes list includes information on sex, marital status and number of children. However, this information proved rather incomplete or too unspecific for the current purpose. Together with a team of student assistants, I therefore collected information on these characteristics drawing on contents from newspaper archives and Web sources. In order to minimize the percentage of missing cases, we were very meticulous in this effort, spending on average roughly two hours search time per listee. The amended part of the data set includes information on listees' number and sex of their biological as well as adoptive and step children.

Of the initial 400 cases, 39 women and 87 men were listed as heirs, and 3 women and 271 men as self-made billionaires. A total of 374 cases remained in the sample after 20 childless billionaires, three cases for which parental status could not be identified, and the only three self-made female billionaires were removed from the data. The latter group was removed because the group size is too small for reasonable subgroup analyses. The remaining sample consists of 37 heiresses, 82 heirs, and 255 self-made, male billionaires. Of the 37 heiresses, 26 inherited their wealth from their parents and 11 from their deceased partner. Of the 82 heirs, 51 inherited their wealth from parents who were themselves self-made billionaires, 30 from parents who were themselves heirs, and one inherited in first generation from a non-family member.

Heiresses who inherited their wealth from their partners should be distinguished from those who inherited from their parents. This distinction is necessary for two reasons: First, it should matter if the male or female partner in a dyad is responsible for achieving wealth, given a strong association between social status and mating preferences [35]. Second and highly relevant with regard to the second hypothesis stated at the end of the previous section, the timing of wealth accumulation is different in the two groups of heiresses: On the one hand, those heiresses who inherited from their parents have become wealthy before their childbearing years. On the other hand, those heiresses who inherited from their partners either have or have not become wealthy before their childbearing years, depending on whether their deceased spouse or partner was a self-made billionaire or heir. If the inheritance comes from an heir, wealth-status was achieved before the childbearing years. Therefore, these women should be treated like those married to a living heir. In contrast, if the inheritance comes from a self-made billionaire, wealth-status may not have been achieved upon conception of at least some of the children of these women. In this case they should be treated like women married or partnered to a living self-made billionaire. Therefore, the 11 heiresses identified above who inherited from their deceased partner should be reassigned accordingly. This leaves the following dyadic constellations that are used in subsequent analyses: 262 self-made billionaires with wives or partners, 86 heirs with wives or partners, and 26 heiresses who inherited from their parents with husbands or partners.

Information on offspring sex is complete for 93.32% of all listees. For an additional 4.28% of listees information on the sex of at least some children is available. This leaves only 2.41% of listees for which the sex of none of their children is known. If we count the number of missing cases as the percentage of children whose sex is unknown we have the sex missing for 4.39% of all recorded children, leaving a total of 1165 valid cases of biological children. Except for the three individuals, for whom parental status could not be identified, the total number of children is known for all cases. This turnout means a considerable improvement in coverage as compared to previous studies in which, using Forbes data from 1982 and 2008, information on offspring sex was missing for more than half of the respective samples of wealthy parents [19], [20].

## Results

To examine whether a TW effect can be found in the sample of billionaires, I used a combination of different methods. First, I conducted a series of binomial tests to compare the proportion of male offspring (p_m_) for the complete sample of billionaires and for specific billionaire subgroups to the corresponding proportion in the general U.S. population. Second, I examined differences in the proportion of male offspring between billionaire subgroups. Third, I conducted a robustness test by repeating the group comparison using a multilevel logistic regression model. In the following, I report the percentages of male offspring for the complete sample of billionaires and for specific subgroups. Sex ratios (SR), defined here as number of males per female, are reported in brackets. The following formula (F1) is used to transform values for the proportion of male offspring into sex ratios:



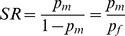
(F1)


**Figure 1:**
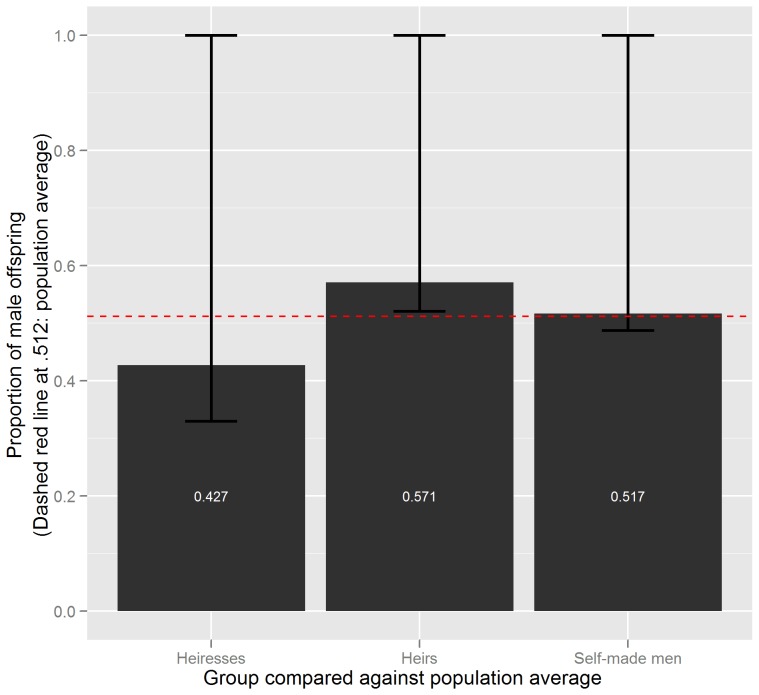
One-sided binomial tests comparing the percentage of male offspring against the population average, by billionaire subgroup. Results of one-sided binomial tests with 95% confidence intervals. The dashed red line indicates the average share of male offspring in the general population (p_m_≈.512, SR = 1.05).

With about 52.4% (SR: 1.10), the percentage of male offspring for the sample of billionaires is just slightly higher than the corresponding percentage in the general US population for the last half century (51.1–51.4%; SR: 1.05–1.06) [36]. Given that this difference is not statistically significant, it speaks against the first hypothesis that billionaires have a higher percentage of male offspring than the general population. However, results differ markedly between billionaire subgroups: Broken down by wealth origin, sex ratios among males are much higher for heirs than for self-made billionaires. Heirs have 57.1% sons (SR: 1.33) on average and self-made billionaires 51.7% (SR: 1.07) (see Table 1 and Figure 1). This difference is consistent with the stated hypothesis on the relative timing of wealth accumulation and childbearing. Given that self-made men make up the majority of the Forbes sample of billionaire parents (255 of 374 cases), it is no surprise that the sex ratio of the general sample is similar to the one of male, self-made billionaires. With 42.7% (SR: 0.74), heiresses have a percentage of male offspring that is considerably lower than that of the general population.

**Table 1:** One-sided binomial tests of percentage of male offspring among billionaire subgroups vs. general population (p_m_≈.512, SR = 1.05).

	p_m_	(SR)	95% CI		p	p_Bonferroni_
all	.524	(1.10)	.500	1.000	.209	–
self-made men	.517	(1.07)	.400	1.000	.400	1.000
heirs	.571	(1.33)	.521	1.000	.027	.081
heiresses	.427	(.74)	.000	.528	.945	1.000

Some, but not all, of the reported differences in this paper are statistically significant. For the comparison with the general US population, I conducted exact binomial tests for the probability of male births. Given that the data are grouped, containing information on multiple children per listee, I calculated an empty logistic regression model with a random intercept only and sex of the child as dependent variable to obtain the intraclass correlation (ICC). The ICC depicts possible within-parent dependencies that can affect the binomial test [37]. However, given a very low ICC of almost zero (ρ ≈ 1.48•10^−12^), there are no dependencies within parents. In other words, the sex of one child is not connected to the sex of another child from the same billionaire. In the following, I thus use regular binomial tests. The first hypothesis suggests a specific direction of the deviation from the population percentage of male offspring [19], [38]. Therefore, the tests are one-sided, testing whether billionaires have a higher-than-average percentage of male offspring. Using a conventional level of statistical significance of .05, only the percentage of male offspring among heirs deviates significantly from the US population average. However, performing a Bonferroni correction for multiple comparisons [cf. 16] leaves all effects insignificant (see Table 1). The comparison between groups in this sample rather than between groups and the population average serves to test the second hypothesis, that is, whether heirs or heiresses have a higher percentage of male offspring than self-made billionaires. The one-sided test for a higher proportion of male offspring among heirs than among self-made billionaires reaches almost statistical significance (p≈.067). But the one-sided test for a higher proportion of male offspring among heiresses than among self-made billionaires does not (p≈.916). Because the initially stated hypotheses are indifferent with regard to the comparison between heirs and heiresses, I conducted a two-sided test for comparing their respective percentages of male offspring. The result is that heiresses have a significantly lower percentage of male offspring than heirs (p_m_≈.427 vs. p_m_≈.571; p≈.035). However, after correcting for multiple comparisons all three tests are insignificant (see Table 2).

**Table 2:** One- or two sided tests of equal proportions between groups for percentage male offspring between groups of billionaires.

group 1	group 2	p_m1_	p_m2_	p	p_Bonferroni_	test direction
heirs	self-made men	.571	.517	.067	.201	group 1>group 2
heirs	heiresses	.571	.427	.035	.105	group 1 = group 2
self-made men	heiresses	.517	.427	.916	1.000	group 1<group 2

As a robustness check, the influence of billionaire status (heiress, heir, self-made man) on offspring sex was also tested using a multilevel logistic regression. Whereas in the pooled model, clustering of multiple births among the same billionaire parents is not accounted for, in the random effects model this is achieved by including parent-level random intercepts. Both models yield the same results (see Table 3), confirming thus the low degree of within-person dependence that was earlier reported with an ICC of almost zero. The log odds (β) obtained in a logistic regression can easily be transformed into odds ratios (e^β^) which correspond to the respective sex ratios (SR) (see F2). The predicted sex ratios from the logistic regression in Table 3 correspond to the ones reported in Table 1: Heirs have a predicted and rounded sex ratio of 1.33, heiresses of .74 (≈ 1.333*.558), and self-made men of 1.07 (≈ 1.333*.804). Whereas neither the sex ratio of self-made men differs significantly from that of heirs (p ≈ .118) nor that of heiresses from that of self-made men (p≈.135), the difference between heiresses and heirs is statistically significant (p≈.027). In a simultaneous coefficient test that corrects for multiple comparisons, the difference between heiresses and heirs remains significant in a one-sided (p≈.032) and almost significant in a two-sided test (p≈.064). The slightly lower p-values may be explained by the higher power of multilevel models [39] – although here that effect should be small, given the extremely low degree of within-parent correlation. Both the coefficient test for the comparison of heiresses and self-made men and the correction for multiple comparisons were conducted using the ‘multcomp’ command in R [40].

**Table 3:** Comparison of pooled and random effects logistic regression analysis on offspring sex (male = 1).

	pooled model				random effects model			
	β	S.E.	SR( = e^β^)	p	β	S.E.	SR( = e^β^)	p
(Intercept)	.288	.121	1.333	.017	.288	.121	1.333	.017
Group (ref: heirs)								
heiresses	−.583	.263	.558	.027	−.583	.263	.558	.027
self-made men	−.219	.140	.804	.118	−.219	.140	.804	.118
Model summary:	N = 1165AIC = 1612.7				N_children_ = 1165, N_parents_ = 364AIC = 1615			



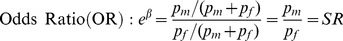
(F2)


## Discussion

The results clearly speak against the first hypothesis and thus against the existence of a TW effect in all subgroups of U.S. billionaires. This finding deviates in part from earlier studies analyzing similar data sets. On the one hand it confirms the result from at least one other study based on Forbes data from 1982 which also reports a null-effect [20]. On the other hand, it stands in clear contrast to the finding from a study based on Forbes data from 2008 which reports a very large TW-consistent finding for the complete male billionaire sample [19]. The mixed pattern may be explained in part by the extremely large share of cases with missing information: in both studies more than half of the respective sample is without information on offspring sex. In the current study, the share of missing values could be considerably reduced. The results are therefore less subject to a selection bias that could result from the higher difficulty to retrieve information on female rather than male offspring.

In the second hypothesis I predicted a higher proportion of male offspring among billionaires who inherited their wealth than among self-made billionaires. This hypothesis is weakly supported, but only for men: Heirs have a much higher share of male offspring than both self-made billionaires and the general population. They have their complete fertility history as wealthy individuals: Every time a new child is conceived by the spouse or partner of an heir, sex composition may be affected by the putative TW mechanism. Self-made billionaires, on the other hand, only have a slightly higher percentage of male offspring than the general population, a difference that is not statistically significant. This is plausible, given that self-made billionaires have at least some of their children at a time when they have not yet achieved high wealth status. In emphasizing the importance of timing of socioeconomic condition in measuring the TW effect, this finding is consistent with research based on animal studies that finds a higher share of TW-consistent results in studies that measure parental condition close or around the time of conception [28]. The difference between heirs and self-made billionaires is, however, not statistically significant when the two groups are compared directly. But heirs, as opposed to self-made billionaires, show a significantly higher share of male offspring than the average U.S. population, a difference that remains slightly above the level of statistical significance after adjustment for multiple comparisons. Previous studies ignore the distinction between self-made billionaires and heirs or find no difference in offspring sex ratios between the two groups which might, again, be due to the selection bias present in those studies [19], [20].

A limitation of the current study is that the timing of wealth accumulation, self-made wealth vs. inheritance, is rather coarse. In fact, among self-made billionaires there is considerable variation in the type of occupations parents hold, including both sons of poor immigrants and sons of parents with high-status jobs like lawyers, doctors, or scientists. There is also considerable variation with regard to the timing of status achievement and childbearing. In fact, the group of self-made billionaires should consist of individuals who achieved high wealth status before they started their fertility history, during their fertility history, and after they completed their fertility history. If future data collection efforts could yield more fine-tuned measurements on these dimensions, more detailed hypotheses on the timing and strength of the TW effect would become possible.

A striking result of the current study, one implied by neither of the two initially stated variants of the TW hypothesis, is that heiresses have a considerably lower percentage of male offspring than heirs, self-made billionaires, and the general population. That is, for women, the observed effect is actually diametrical to the prediction made on the basis of the TW hypothesis. The difference in the proportion of male offspring between heiresses and heirs is, in fact, the highest difference observed in this study. Despite the large size of the effect and due to the low group size of 26 heiresses, however, it is only statistically significant when not controlling for multiple comparisons. Once this correction is applied, the difference remains only slightly above statistical significance in the logistic regression model. Future research should test whether this finding can be replicated with larger samples of elite women. Also, future samples should be extended to include a higher share of women who are self-made billionaires. In the current analysis, the difference between heiresses and self-made women could not be tested.

These objections notwithstanding: What could drive this distinct finding for men and for women? A clue to an answer may be provided by the literature on the effect of various stressors on reducing the percentage of male offspring [13], [41]–[44], including occupational stress [38]. If stress and status both affect sex composition, at least two mechanisms may interact and confound each other in producing sex ratios in population subgroups. Could it be that heiresses, partners and spouses of self-made men, and those of male heirs have, on average, different work and career patterns and are thus exposed to various degrees of occupational stress? Specifically, heiresses may be more likely than the spouses of male billionaires to hold stressful leadership positions in companies they inherited from their parents. In order to disentangle the potential interplay of these different mechanisms, future research should pay attention to the occupational status not only of focal individuals but also their spouses and partners. Furthermore, a more detailed analysis is necessary to test their robustness due to other factors that have been pointed out in the literature as affecting sex ratios. U.S. population data, if broken down by mother's age at birth, ethnic origin, and live birth order reveals considerable variation in sex ratios [36]. Also partnership status has been shown to affect sex ratios [45] which could matter with regard to the billionaire sample if partnership patterns differ significantly between subgroups of billionaires.

In sum, the current study contributes importantly to research on the upper limits of the TW effect in humans. First, it shows that no TW effect could be discerned in the full sample of U.S. billionaires. Second, it provides estimates that are sensitive to the timing of maternal condition and conception of a child. Third, it illustrates that distinctive mechanisms may be at play and produce diametrical effects, depending on whether the focal elite individual is male or female. More detailed data on the occupational and fertility histories of wealthy individuals and their partners are necessary to shed more light on how the TW mechanism operates. Promising sources of data are population registers: these include in-depth information on occupational and fertility histories for a very large number of individuals and their partners [46]. Although effects found for the general population are usually very small [17], [18], a higher degree of detail in the measurement and timing of occupational status, wealth and income, as well as fertility, may yield larger effects even in samples of the general population. This is demonstrated in studies on stress- and nutritional effects on sex ratios where the reported effects are considerably higher, when the examined cause occurs closer to the time of conception [42], [47]. A more detailed measurement therefore could also benefit research on the TW effect among human elite samples and allow one to disentangle the various mechanisms that are at play in shaping sex composition in humans.
